# Chlorido{2-[1-(2-pyridylmethyl­imino)eth­yl]pyrrolato-κ^3^ *N*,*N*′,*N*′′}copper(II)

**DOI:** 10.1107/S1600536808006934

**Published:** 2008-03-14

**Authors:** Rongqing Li, Pusu Zhao, Guodong Tang, Yujia Tao

**Affiliations:** a Jiangsu Key Laboratory for the Chemistry of Low-Dimensional Materials, Huaiyin Teachers College, Huai’an 223300, Jiangsu Province, People’s Republic of China

## Abstract

The potential tridentate Schiff base ligand 2-[1-(2-pyridyl­methyl­imino)eth­yl]pyrrole (H*L*) was synthesized from the condensation of 2-acetyl­pyrrole with 2-amino­methyl­pyridine. The title compound, [Cu(C_12_H_12_N_3_)Cl], was synthesized from H*L* and copper(II) chloride using triethyl­amine as a base to deprotonate the pyrrole NH group. The title compound is a monomer and the central copper(II) ion is bound to three N atoms of the deprotonated tridentate ligand and to one chloride ion in a square-planar N_3_Cl coordination.

## Related literature

For related literature, see: Bertrand & Kirkwood (1972 ▶); Brooker & Carter (1995 ▶); Brown *et al.* (1988 ▶); Garland *et al.* (1996 ▶).
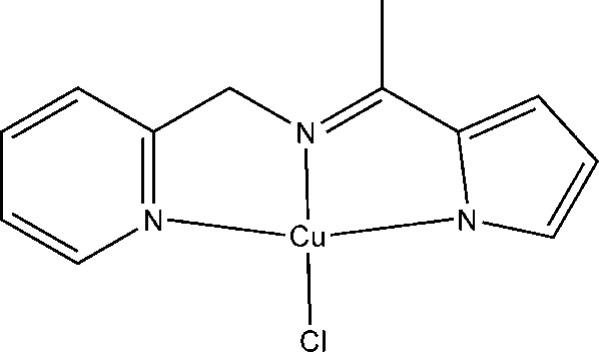


## Experimental

### 

#### Crystal data

[Cu(C_12_H_12_N_3_)Cl]
*M* *_r_* = 297.24
Monoclinic, 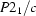

*a* = 8.830 (2) Å
*b* = 7.2806 (15) Å
*c* = 18.750 (4) Å
β = 100.448 (4)°
*V* = 1185.4 (4) Å^3^
*Z* = 4
Mo *K*α radiation
μ = 2.05 mm^−1^
*T* = 213 (2) K
0.24 × 0.18 × 0.16 mm

#### Data collection

Bruker SMART APEX CCD diffractometer
Absorption correction: multi-scan (*SADABS*; Bruker, 2000 ▶) *T*_min_ = 0.642, *T*_max_ = 0.719
11059 measured reflections
2164 independent reflections
1840 reflections with *I* > 2σ(*I*)
*R*_int_ = 0.045

#### Refinement

*R*[*F* ^2^ > 2σ(*F* ^2^)] = 0.049
*wR*(*F* ^2^) = 0.124
*S* = 1.05
2164 reflections
156 parameters
H-atom parameters constrained
Δρ_max_ = 0.38 e Å^−3^
Δρ_min_ = −0.43 e Å^−3^

### 

Data collection: *SMART* (Bruker, 2000 ▶); cell refinement: *SAINT* (Bruker, 2000 ▶); data reduction: *SAINT*; program(s) used to solve structure: *SHELXTL* (Sheldrick, 2008 ▶); program(s) used to refine structure: *SHELXTL*; molecular graphics: *SHELXTL*; software used to prepare material for publication: *SHELXTL*.

## Supplementary Material

Crystal structure: contains datablocks I, global. DOI: 10.1107/S1600536808006934/hg2384sup1.cif

Structure factors: contains datablocks I. DOI: 10.1107/S1600536808006934/hg2384Isup2.hkl

Additional supplementary materials:  crystallographic information; 3D view; checkCIF report

## Figures and Tables

**Table:** 

Cu1—N1	1.943 (4)
Cu1—N2	1.956 (3)
Cu1—N3	2.006 (3)
Cu1—Cl1	2.2319 (12)

**Table:** 

N1—Cu1—N2	81.98 (16)
N1—Cu1—N3	163.31 (15)
N2—Cu1—N3	81.33 (14)
N1—Cu1—Cl1	98.29 (12)
N2—Cu1—Cl1	178.47 (10)
N3—Cu1—Cl1	98.40 (10)

**Fig. 1.**
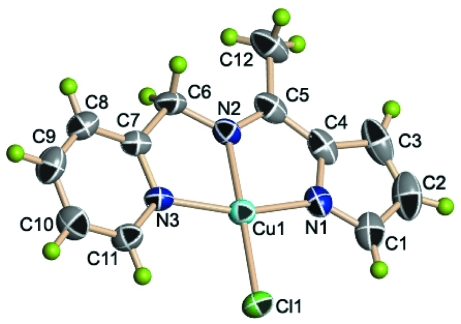
View of the title compound [CuLCl]. Displacement ellipsoids for non-H atoms are drawn at the 30% probability level.

**Fig. 2.**
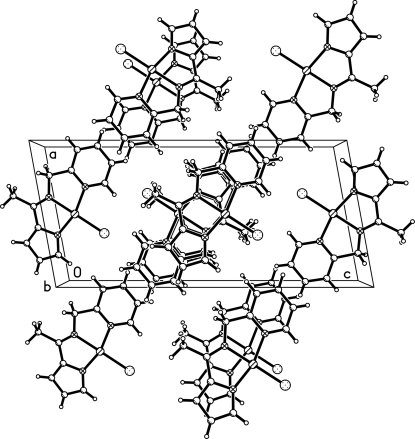
View of the crystal packing along b axis of the unit cell of the monomeric title complex [CuLCl].

## supplementary crystallographic information

### Comment

Many efforts have been made to investigate complexes of wide range of acyclic Schiff base ligands, in particular the pyridine containing systems. However, Much less interest has been attracted in complexes of pyrrole-analogues of such ligands. Recently, our attention has been turned to the copper(II) chemistry of N_3_ tridentate Schiff base ligands. Ligand *L*^-^, the deprotonated form of H*L*, used for the synthesis of the title complex is of this type.

The structure of the title compound consists of isolated neutral monomeric [Cu*L*Cl] molecules (Fig. 1). The copper(II) ion is bound to three nitrogen atoms (comprised of one deprotonated pyrrole nitrogen donor, one pyridine nitrogen donor and one imine nitrogen donor) of the deprotonated tridentate ligand and to one chloride ion, giving an N_3_Cl coordination sphere. The geometry of the coordination polyhedron around the copper(II) ion is square planar (Σangles at Cu = 360.0°). Of the three Cu—N bond distances, the shortest one occurs between the copper atom and the deprotonated negatively charged pyrrole nitrogen atom (N1—Cu1) and the longest one forms between the copper atom and the pyridine nitrogen donor which is *trans* to the pyrrole nitrogen (Cu1—N3). The two *cis* N—Cu—N angles are very similar and are both smaller than 90°. This is as expected as both of the angles are part of five-membered, pyrrole-imine or pyridine-imine, chelate rings. The two *cis* N—Cu—Cl angles are similar to one another but are both bigger than a right angle. The Cu—N1 (pyrrole nitrogen) bond distance is very similar to that reported for the related copper(II) complexes (Bertrand & Kirkwood, 1972; Brooker & Carter, 1995). Cu—N (pyridine nitrogen) bonds are usually 2.00–2.05 Å long (Brown *et al.*, 1988; Garland *et al.*, 1996), so the Cu—N3 (pyridine nitrogen) distance in this complex [2.007 (4) Å] is normal.

### Experimental

Ligand H*L* was synthesized from the condensation of 2-acetylpyrrole with 2-aminomethylpyridine.

To a solution of Ligand H*L* (0.375 mmol) in methanol (5 ml) was added triethylamine (0.385 mmol) in methanol (5 ml). To this resulting solution was added a green solution of copper(II) chloride dihydrate (0.375 mmol) in methanol (5 ml), over which time a precipitate formed. The resulting mixture was stirred for 3 hr after which the green solid was collected by filtration, washed with methanol and dried in *vacuo*. Yield: 0.093 g (80% based on copper(II) chloride used). Single crystals of [Cu*L*Cl] were obtained by vapour diffusion of diethyl ether into a dichloromethane solution. Analysis: found C 48.74, H 3.97, N 14.18; calculated for C_12_H_12_N_3_CuCl C 48.49, H 4.07, N, 14.14%. IR: ν, cm^-1^, 1601 (C?N).

### Refinement

Hydrogen atoms were positioned geometrically and refined using a riding model, with C—H bonds = 0.93–0.97 Å and with *U*_iso_ (H) = 1.2*U*_eq_ (C) [1.5*U*_eq_ (C) for the methyl group].

### Crystal data

**Table:**

[Cu(C_12_H_12_N_3_)Cl]	*F*_000_ = 604
*M**_r_* = 297.24	*D*_x_ = 1.666 Mg m^−^^3^
Monoclinic, *P*2_1_/*c*	Mo *K*α radiation λ = 0.71070 Å
Hall symbol: -P 2ybc	Cell parameters from 3740 reflections
*a* = 8.830 (2) Å	θ = 2.1–25.6º
*b* = 7.2806 (15) Å	µ = 2.05 mm^−^^1^
*c* = 18.750 (4) Å	*T* = 213 (2) K
β = 100.448 (4)º	Block, green
*V* = 1185.4 (4) Å^3^	0.24 × 0.18 × 0.16 mm
*Z* = 4	

### Data collection

**Table:**

Bruker SMART APEX CCD diffractometer	2164 independent reflections
Radiation source: sealed tube	1840 reflections with *I* > 2σ(*I*)
Monochromator: graphite	*R*_int_ = 0.045
*T* = 213(2) K	θ_max_ = 25.4º
φ and ω scans	θ_min_ = 3.0º
Absorption correction: multi-scan(SADABS; Bruker, 2000)	*h* = −10→10
*T*_min_ = 0.642, *T*_max_ = 0.719	*k* = −7→8
11059 measured reflections	*l* = −22→22

### Refinement

**Table:**

Refinement on *F*^2^	Secondary atom site location: difference Fourier map
Least-squares matrix: full	Hydrogen site location: inferred from neighbouring sites
*R*[*F*^2^ > 2σ(*F*^2^)] = 0.049	H-atom parameters constrained
*wR*(*F*^2^) = 0.124	*w* = 1/[σ^2^(*F*_o_^2^) + (0.06*P*)^2^ + 1.99*P*] where *P* = (*F*_o_^2^ + 2*F*_c_^2^)/3
*S* = 1.05	(Δ/σ)_max_ < 0.001
2164 reflections	Δρ_max_ = 0.38 e Å^−^^3^
156 parameters	Δρ_min_ = −0.43 e Å^−^^3^
Primary atom site location: structure-invariant direct methods	Extinction correction: none

### Special details

**Table:**

Geometry. All e.s.d.'s (except the e.s.d. in the dihedral angle between two l.s. planes) are estimated using the full covariance matrix. The cell e.s.d.'s are taken into account individually in the estimation of e.s.d.'s in distances, angles and torsion angles; correlations between e.s.d.'s in cell parameters are only used when they are defined by crystal symmetry. An approximate (isotropic) treatment of cell e.s.d.'s is used for estimating e.s.d.'s involving l.s. planes.
Refinement. Refinement of *F*^2^ against ALL reflections. The weighted *R*-factor *wR* and goodness of fit *S* are based on *F*^2^, conventional *R*-factors *R* are based on *F*, with *F* set to zero for negative *F*^2^. The threshold expression of *F*^2^ > σ(*F*^2^) is used only for calculating *R*-factors(gt) *etc*. and is not relevant to the choice of reflections for refinement. *R*-factors based on *F*^2^ are statistically about twice as large as those based on *F*, and *R*- factors based on ALL data will be even larger.

### Fractional atomic coordinates and isotropic or equivalent isotropic displacement parameters (Å^2^)

**Table:**

	*x*	*y*	*z*	*U*_iso_*/*U*_eq_	
Cu1	0.50155 (6)	0.79633 (7)	0.42653 (3)	0.0312 (2)	
Cl1	0.64180 (13)	0.80713 (16)	0.33884 (6)	0.0431 (3)	
N1	0.6561 (4)	0.6909 (5)	0.5025 (2)	0.0378 (9)	
N2	0.3797 (4)	0.7939 (5)	0.50388 (19)	0.0331 (8)	
N3	0.3055 (4)	0.9028 (5)	0.37105 (17)	0.0303 (8)	
C1	0.8039 (6)	0.6345 (6)	0.5150 (3)	0.0479 (12)	
H1	0.8685	0.6360	0.4810	0.057*	
C2	0.8452 (7)	0.5741 (8)	0.5857 (3)	0.0648 (16)	
H2	0.9412	0.5291	0.6073	0.078*	
C3	0.7176 (7)	0.5928 (7)	0.6184 (3)	0.0593 (15)	
H3	0.7107	0.5626	0.6659	0.071*	
C4	0.6022 (6)	0.6658 (6)	0.5659 (2)	0.0423 (11)	
C5	0.4452 (6)	0.7218 (6)	0.5646 (2)	0.0413 (11)	
C6	0.2223 (5)	0.8562 (6)	0.4865 (2)	0.0374 (10)	
H6A	0.2082	0.9629	0.5156	0.045*	
H6B	0.1534	0.7601	0.4970	0.045*	
C7	0.1863 (5)	0.9048 (6)	0.4073 (2)	0.0327 (9)	
C8	0.0406 (5)	0.9525 (7)	0.3731 (3)	0.0434 (12)	
H8	−0.0410	0.9485	0.3982	0.052*	
C9	0.0156 (6)	1.0060 (7)	0.3019 (3)	0.0514 (13)	
H9	−0.0829	1.0374	0.2783	0.062*	
C10	0.1382 (6)	1.0128 (7)	0.2655 (3)	0.0471 (12)	
H10	0.1249	1.0534	0.2178	0.057*	
C11	0.2804 (5)	0.9580 (6)	0.3018 (2)	0.0371 (10)	
H11	0.3628	0.9593	0.2771	0.045*	
C12	0.3689 (7)	0.6982 (7)	0.6292 (3)	0.0551 (14)	
H12A	0.2995	0.7987	0.6315	0.083*	
H12B	0.4458	0.6960	0.6725	0.083*	
H12C	0.3124	0.5849	0.6249	0.083*	

### Atomic displacement parameters (Å^2^)

**Table:**

	*U*^11^	*U*^22^	*U*^33^	*U*^12^	*U*^13^	*U*^23^
Cu1	0.0315 (3)	0.0342 (3)	0.0282 (3)	−0.0016 (2)	0.0058 (2)	0.0003 (2)
Cl1	0.0374 (6)	0.0497 (7)	0.0463 (7)	0.0016 (5)	0.0183 (5)	0.0013 (5)
N1	0.038 (2)	0.032 (2)	0.039 (2)	−0.0039 (16)	−0.0028 (17)	0.0009 (16)
N2	0.044 (2)	0.0289 (18)	0.0278 (19)	−0.0009 (16)	0.0090 (16)	−0.0006 (15)
N3	0.0331 (18)	0.0318 (18)	0.0275 (18)	−0.0058 (16)	0.0094 (15)	−0.0027 (15)
C1	0.043 (3)	0.037 (3)	0.057 (3)	−0.003 (2)	−0.010 (2)	−0.007 (2)
C2	0.065 (4)	0.051 (3)	0.064 (4)	0.008 (3)	−0.028 (3)	−0.009 (3)
C3	0.094 (4)	0.035 (3)	0.036 (3)	0.005 (3)	−0.022 (3)	−0.002 (2)
C4	0.066 (3)	0.025 (2)	0.032 (2)	−0.003 (2)	−0.003 (2)	−0.0036 (18)
C5	0.066 (3)	0.025 (2)	0.034 (2)	−0.008 (2)	0.010 (2)	−0.0033 (19)
C6	0.045 (3)	0.036 (2)	0.037 (2)	−0.005 (2)	0.021 (2)	−0.001 (2)
C7	0.038 (2)	0.031 (2)	0.031 (2)	−0.0075 (19)	0.0108 (18)	−0.0060 (19)
C8	0.033 (2)	0.050 (3)	0.048 (3)	−0.004 (2)	0.011 (2)	−0.005 (2)
C9	0.036 (3)	0.057 (3)	0.056 (3)	0.006 (2)	−0.003 (2)	−0.009 (3)
C10	0.053 (3)	0.053 (3)	0.031 (2)	−0.003 (2)	−0.002 (2)	−0.002 (2)
C11	0.034 (2)	0.047 (3)	0.031 (2)	−0.004 (2)	0.0074 (19)	−0.005 (2)
C12	0.094 (4)	0.042 (3)	0.032 (3)	−0.005 (3)	0.018 (3)	0.004 (2)

### Geometric parameters (Å, °)

**Table:**

Cu1—N1	1.943 (4)	C4—C5	1.441 (7)
Cu1—N2	1.956 (3)	C5—C12	1.499 (7)
Cu1—N3	2.006 (3)	C6—C7	1.503 (6)
Cu1—Cl1	2.2319 (12)	C6—H6A	0.9700
N1—C1	1.347 (6)	C6—H6B	0.9700
N1—C4	1.370 (6)	C7—C8	1.374 (6)
N2—C5	1.291 (6)	C8—C9	1.369 (7)
N2—C6	1.442 (6)	C8—H8	0.9300
N3—C11	1.339 (5)	C9—C10	1.381 (7)
N3—C7	1.354 (5)	C9—H9	0.9300
C1—C2	1.381 (7)	C10—C11	1.374 (6)
C1—H1	0.9300	C10—H10	0.9300
C2—C3	1.384 (8)	C11—H11	0.9300
C2—H2	0.9300	C12—H12A	0.9600
C3—C4	1.388 (7)	C12—H12B	0.9600
C3—H3	0.9300	C12—H12C	0.9600
			
N1—Cu1—N2	81.98 (16)	C4—C5—C12	121.7 (4)
N1—Cu1—N3	163.31 (15)	N2—C6—C7	108.7 (3)
N2—Cu1—N3	81.33 (14)	N2—C6—H6A	110.0
N1—Cu1—Cl1	98.29 (12)	C7—C6—H6A	110.0
N2—Cu1—Cl1	178.47 (10)	N2—C6—H6B	110.0
N3—Cu1—Cl1	98.40 (10)	C7—C6—H6B	110.0
C1—N1—C4	106.6 (4)	H6A—C6—H6B	108.3
C1—N1—Cu1	141.0 (4)	N3—C7—C8	121.0 (4)
C4—N1—Cu1	112.4 (3)	N3—C7—C6	116.7 (4)
C5—N2—C6	125.9 (4)	C8—C7—C6	122.3 (4)
C5—N2—Cu1	116.0 (3)	C9—C8—C7	119.9 (4)
C6—N2—Cu1	117.8 (3)	C9—C8—H8	120.0
C11—N3—C7	118.5 (4)	C7—C8—H8	120.0
C11—N3—Cu1	126.5 (3)	C8—C9—C10	119.3 (5)
C7—N3—Cu1	114.8 (3)	C8—C9—H9	120.3
N1—C1—C2	110.1 (5)	C10—C9—H9	120.3
N1—C1—H1	125.0	C11—C10—C9	118.2 (5)
C2—C1—H1	125.0	C11—C10—H10	120.9
C1—C2—C3	107.4 (5)	C9—C10—H10	120.9
C1—C2—H2	126.3	N3—C11—C10	122.9 (4)
C3—C2—H2	126.3	N3—C11—H11	118.6
C2—C3—C4	105.9 (5)	C10—C11—H11	118.6
C2—C3—H3	127.0	C5—C12—H12A	109.5
C4—C3—H3	127.0	C5—C12—H12B	109.5
N1—C4—C3	109.9 (5)	H12A—C12—H12B	109.5
N1—C4—C5	115.6 (4)	C5—C12—H12C	109.5
C3—C4—C5	134.5 (5)	H12A—C12—H12C	109.5
N2—C5—C4	113.9 (4)	H12B—C12—H12C	109.5
N2—C5—C12	124.3 (5)		
